# Cardiorespiratory Fitness Assessed by Direct VO₂peak Measurement in Adolescent and Young Adult Males With Developmental Disorders

**DOI:** 10.7759/cureus.90024

**Published:** 2025-08-13

**Authors:** Ayako Satonaka, Michio Wachi, Nobuharu Suzuki

**Affiliations:** 1 Department of Medical Sciences, Aichi Syukutoku University, Aichi, JPN; 2 Department of Physical Therapy, Bukkyo University, Kyoto, JPN

**Keywords:** adolescent and young adults, cardiorespiratory fitness, developmental disorders, maximal exercise testing, peak oxygen uptake

## Abstract

Introduction

Individuals with developmental disorders (DD) often exhibit reduced levels of daily physical activity, contributing to diminished cardiorespiratory fitness. While field-based tests such as the 20-meter shuttle run and six-minute walk test are commonly used for indirect assessment, few studies have employed direct measurement of peak oxygen uptake (VO_2_peak), the gold standard for evaluating aerobic capacity. This study aimed to evaluate VO_2_peak using a maximal exercise test in adolescent and young adult males with developmental disorders.

Methods

A cross-sectional study was conducted with 41 male participants aged 15-20 years with diagnosed developmental disorders. After excluding individuals with BMI >30, 37 participants completed a ramp protocol maximal exercise test on a cycle ergometer. VO_2_peak was measured using breath-by-breath gas analysis. Values were compared to established normative data for healthy Japanese males of similar age.

Results

The mean absolute VO_2_peak was 2209.9 ± 458 mL/min, and the weight-adjusted VO_2_peak was 38.1 ± 6.8 mL/min/kg. Only 6 out of 37 participants fell within the normative range (95% CI: 44.2-60.2 mL/min/kg), indicating significantly reduced cardiorespiratory fitness in this population. This study is among the first to report directly measured VO_2_peak using a ramp protocol in individuals with developmental disorders.

Conclusion

Adolescent and young adult males with developmental disorders demonstrated substantially lower VO_2_peak values compared to their typically developing peers. These findings highlight the need for targeted physical activity interventions and provide reference data for future research in this under-investigated population.

## Introduction

Individuals with developmental disorders tend to engage in lower levels of daily physical activity compared to their typically developing peers [1-4]. As such, there is growing interest in the effects of exercise interventions for this population. In particular, several studies have suggested that aerobic exercise may help alleviate core symptoms of neurodevelopmental disorders and improve social behavior [5-13]. For example, Ferreira et al. conducted a systematic review and meta-analysis involving children with autism spectrum disorder (ASD), demonstrating that physical activity is an effective means of reducing the frequency of stereotyped behaviors in children with ASD [11]. Bremer et al. reviewed behavioral changes following exercise interventions in children under 16 years with ASD, reporting that interventions such as jogging, horseback riding, martial arts, swimming, and yoga/dance may positively impact stereotyped behaviors, socioemotional functioning, cognitive function, and attention [8]. Hattabi et al. found that a 12-week swimming-based recreational program in children with ADHD yielded measurable improvements across inattentiveness, hyperactivity, and impulsivity domains [13]. These findings collectively suggest that aerobic exercise can be beneficial for children and adolescents with developmental disorders and underscore the importance of encouraging their participation in such activities.

Cardiorespiratory endurance is expected to improve when aerobic exercise is performed with appropriately prescribed intensity, duration, and frequency [14,15]. Individuals with developmental disorders often exhibit low cardiorespiratory endurance and increased susceptibility to fatigue, likely resulting from chronically low levels of physical activity and limited participation in aerobic exercise [16,17].

To date, the assessment of cardiorespiratory fitness in children and adolescents with developmental disorders has largely relied on convenient, field-based indirect tests such as the 20-meter shuttle run test and the six-minute walk test [18-22]. Wouters et al. noted the challenges of measuring physical fitness in children and adolescents with intellectual disabilities (ID) and conducted a systematic review of field-based fitness assessments in this population [21]. While some studies demonstrated adequate reliability and validity, they concluded that field-based physical fitness tests may not be suitable for children and adolescents with intellectual disabilities across all age groups and severity levels [21]. Although these indirect field-based assessments offer practical advantages due to their ease of implementation, they have limitations in terms of reproducibility and accuracy in capturing maximal capacity [18-22]. In contrast, studies employing direct physiological measures such as peak oxygen uptake (VO_2_peak) remain scarce [18-23]. VO_2_peak is recognized as the gold standard for assessing cardiorespiratory fitness, providing a quantitative and highly reliable indicator of aerobic capacity [14,24].

The aim of the present study was to evaluate the cardiorespiratory fitness of adolescent and young adult males with developmental disorders using direct measurement of VO_2_peak via a maximal exercise test. Although detailed diagnostic criteria were not accessible to the researchers due to institutional privacy policies, participant eligibility was verified by both the referring physicians and the in-house medical physicians and physical therapists. This study aims to fill a gap in the literature by directly measuring VO_2_peak in this population, providing baseline data to guide future interventions.

## Materials and methods

Study design

This study employed a cross-sectional observational design aimed at evaluating VO_2_peak as an indicator of cardiorespiratory endurance in late-adolescent and young adult males with developmental disorders. Participants underwent a maximal exercise test to obtain a direct and quantitative assessment of VO_2_peak. No intervention was implemented; the primary objective was to evaluate physical fitness at a single point in time.

Participants

The study included 41 adolescent and young adult males aged 15 to 20 years with developmental disorders who were capable of independent daily living. All participants were enrolled in Haruhidai Vocational Training School at the Aichi Human Service Center, an institution designed to support youths aiming for employment, and were residing in the school’s dormitory. Their educational backgrounds varied; while some had graduated from regular junior or senior high schools, not all had attended special education schools. All participants had documented diagnoses of developmental disorders issued by licensed physicians at external medical institutions. While the specific diagnostic criteria were not disclosed to the authors, participant eligibility for the cardiopulmonary exercise test was reconfirmed by the attending physician and a licensed physical therapist affiliated with this study. Inclusion criteria required participants to be able to walk independently and to have been medically cleared to safely perform a maximal exercise test. The study procedures and purpose were explained to both participants and their guardians in both written and verbal form. Written informed consent was obtained from all participants and/or their legal guardians after sufficient explanation and understanding.

This study was approved by the Ethical Review Committee of the Institute of Developmental Research, Aichi Human Service Center (approval number: 13-02).

Measurements

The measurement of VO_2_peak followed procedures described in a previously published study [25]. All assessments were conducted in a climate-controlled laboratory maintained at 22°C and 40% relative humidity. Participants wore light clothing and were provided with a standardized orientation explaining the study's purpose and procedures prior to testing. VO_2_peak measurements were conducted one to two months after the participants entered the dormitory. This timing was selected to account for the potential influence of the dormitory lifestyle, which included daily weekday activities such as stretching exercises, jogging, and vocational training.

Height and body weight were measured using standard anthropometric methods [26]. On the day of testing, a physician assessed each participant’s health status; testing was postponed for individuals presenting with any physical complaints. Electrocardiogram (ECG) electrodes were attached to the chest, and a blood pressure cuff was placed on the left upper arm using an automatic ECG and blood pressure monitor (model BP-308, Nippon Colin Co., Japan). Heart rate was continuously recorded using a sports heart rate monitor (model S810i, Polar Co., Finland).

To assess VO_2_peak during the maximal exercise test, a face mask with an air cushion seal was selected and fitted to each participant based on facial size. The mask was secured using a specialized rubber strap, and air leakage was checked and corrected before testing. Expired gas analysis was performed breath-by-breath using an automated gas analyzer (Aeromonitor model AE-310s, Minato Medical Science Co., Japan) throughout the exercise test.

Maximal exercise test protocol

The maximal exercise test was conducted using a cycle ergometer (Aerobike model 75X, Combi Co., Japan). A ramp protocol was employed, beginning with a two-minute warm-up at 20 watts, followed by a ramped increase in power output at a rate of 20 watts/minute. Participants were instructed to maintain a constant pedaling cadence of approximately 60 revolutions per minute (rpm). Each participant continued exercising until they reached voluntary exhaustion and met the predefined termination criteria.

The termination criteria were as follows: (1) A plateau in oxygen uptake (VO_2_) despite increased workload, or (2) a respiratory exchange ratio (RER) equal to or greater than 1.1.

During the exercise test, a physician continuously monitored the electrocardiogram (ECG) and performed repeated blood pressure measurements.

Statistical analysis

The mean and standard deviation of measured peak oxygen uptake (VO_2_peak) were calculated. Additionally, relative VO_2_peak values, which are VO_2_peak values normalized to body weight (mL/min/kg), were compared with existing reference values for Japanese males reported in a previous study [27].

All collected personal data were anonymized and used solely for statistical analysis and reporting of the research results. All statistical analyses were performed using IBM SPSS Statistics for Windows (version 27.0; IBM Corp., Armonk, NY, USA).

## Results

Table 1 summarizes the physical characteristics of the participants. After excluding individuals with a BMI over 30 and those who did not reach VO_2_peak based on test criteria, data from 37 adolescent and young adult males aged 15 to 20 years (mean age 17.3 ± 1.6 years) were included in the analysis. Their mean height was 167.0 ± 6.4 cm, mean weight was 58.2 ± 10.8 kg, and mean BMI was 20.8 ± 3.0 kg/m².

**Table 1:** Physical characteristics of participants

	Age (years old)	Height (cm)	Weight (kg)	BMI (kg/m²)
mean±SD (range) N	17.3 ± 1.6 (15.2 – 20.0) 37	165.6 ± 10.7 (138.6 – 193.5) 37	58.9 ± 11.9 (41.0 – 80.5) 37	21.4 ± 3.5 (16.8 – 27.8) 37

Table 2 presents the primary outcomes of the maximal exercise test. The mean absolute VO_2_peak was 2209.9 ± 458.0 mL/min (range: 1185.8-3384.3 mL/min), and the mean relative VO_2_peak (normalized for body weight) was 38.1 ± 6.8 mL/min/kg (range: 20.10-52.88 mL/min/kg). The 95% confidence interval for relative VO_2_peak was 35.8-40.4 mL/min/kg.

**Table 2:** Results of peak oxygen uptake VO_2_peak: peak oxygen uptake

Variable	Mean ± SD (range)	N	95% CI
Absolute VO_2_peak (mL/min)	2209.9 ± 458 (1185.8–3384.3)	37	2057.1 – 2362.6
Relative VO_2_peak (mL/min/kg)	38.1 ± 6.8 (20.10 – 52.88)	37	35.8 – 40.4

When compared with previously established reference values for healthy Japanese males aged 10-19 years (mean 52.2 mL/min/kg, 95% CI: 44.2-60.2 mL/min/kg) [27], which closely matched the age range of our study participants, only six of the 37 participants fell within the reference CI range. The remaining 31 exhibited relative VO_2_peak values below the lower bound of the reference range.

## Discussion

​​​​​​In this study, we directly measured peak oxygen uptake (VO_2_peak) as an indicator of cardiorespiratory endurance using a maximal exercise test in 37 adolescent and young adult males aged 15 to 20 years with developmental disorders. The results demonstrated that both absolute and relative VO_2_peak values were lower than the reference values for typically developing males in the same age group. Only five of the 37 participants exhibited VO_2_peak values within the reference range, clearly indicating a tendency toward reduced cardiorespiratory fitness in this population. Importantly, this study is among the few that have employed direct gas analysis to measure VO_2_peak in this population, rather than relying on indirect field-based assessments. These findings provide a valuable foundation for future empirical investigations.

Although the present study targeted adolescent and young adult males aged 15 to 20 years with developmental disorders, the reference values for typically developing peers were derived from previously published literature [27]. The meta-analysis by Akiyama et al., which served as the reference source, included both direct VO_2_peak and maximal oxygen uptake (VO_2_max) and indirect measurement methods [27]. Notably, one large-scale study [28] accounted for approximately 71% of the total sample size (55,521 out of 78,714 participants), potentially exerting a substantial influence on the reported means and standard deviations. In that particular study [28], VO_2_peak was estimated using a submaximal exercise protocol (an indirect method), whereas the current study employed direct gas analysis during exhaustive maximal exercise testing. Therefore, caution is warranted when making direct comparisons between the two datasets. Nevertheless, even with this methodological consideration in mind, the mean relative VO_2_peak observed in our participants (38.1 ± 6.8 mL/min/kg) is substantially lower than the average value reported for healthy Japanese males of similar age using cycle ergometer testing (mean: 52.2 mL/min/kg, 95% CI: 44.2-60.2 mL/min/kg) [27].

Previous studies assessing cardiorespiratory endurance in individuals with intellectual and developmental disorders have predominantly employed field-based tests, such as the 20-meter shuttle run and the six-minute walk test [18-22]. While these methods are convenient and widely applicable in educational settings, they provide only indirect estimates of VO_2_max [21]. Only a limited number of previous studies have measured VO_2_peak using direct methods [23,29].

Silman et al. used a continuous and incremental cycle ergometer protocol to compare VO_2_peak between children aged 12-13 years with and without Developmental Coordination Disorder (DCD) [23]. They found that the DCD group had significantly lower VO_2_peak (mean: 35.0 mL/kg/min) than the non-DCD group (mean: 42.9 mL/kg/min). They have pointed out that field-based tests are susceptible to motivational and environmental influences, particularly in children with DCD, where such factors may significantly affect measurement accuracy [23]. Wu et al. conducted a study in Taiwan comparing cardiorespiratory fitness and endurance between children aged nine to 11 years with DCD and typically developing (TD) peers [29]. Cardiorespiratory fitness was assessed using the Bruce treadmill protocol in a maximal exercise test to determine VO_2_peak [29]. The results showed that the DCD group had significantly lower VO_2_peak (39.7 ± 6.6 mL/kg/min) than the TD group (47.6 ± 6.1 mL/kg/min; p < 0.001) [29]. Furthermore, they compared VO_2_peak with 800m running time (minutes) and concluded that 800m running time had a weak correlation with VO_2_peak, and that its effectiveness was limited, especially in the DCD group [29].

In contrast, the present study employed a maximal exercise test (the gold standard for evaluating cardiorespiratory fitness) and measured VO_2_peak under conditions of volitional exhaustion in adolescent and young adult males with developmental disorders. To our knowledge, this is the first study to report directly measured VO_2_peak values obtained through a ramp protocol using a cycle ergometer in adolescent males with developmental disorders. As such, our findings may serve as a useful reference for designing future research and developing targeted interventions for this population.

The participants in this study were adolescent and young adult males aged 15 to 20 years with developmental disorders; however, not all of them were diagnosed with DCD. Despite this, our findings were consistent with previous studies that specifically targeted children with DCD [23,29], suggesting that reduced cardiorespiratory fitness in this population may not be solely attributable to motor coordination impairments. Rather, it is likely to reflect chronically low levels of physical activity and limited opportunities for endurance training among individuals with developmental disorders. Therefore, future efforts should focus on promoting daily physical activity and creating opportunities for endurance-based exercise. For example, providing structured physical activities such as play-based exercise, dance, or swimming programs tailored for children with developmental disorders may be effective in improving their aerobic fitness.

Limitations

This study has several limitations. First, the participants were limited to males enrolled in a specific vocational training school, which restricts the generalizability of the findings. In addition, the comparison group was based on normative values from the literature rather than a concurrently assessed control group of typically developing individuals. Furthermore, differences in the methodologies used to measure VO_2_peak across studies necessitate cautious interpretation of the results. Additionally, while the VO_2_peak assessments were conducted one to two months after dormitory admission, this time period may not strictly reflect a baseline condition. However, based on well-established exercise physiology principles, meaningful improvements in aerobic capacity generally require more than two months of consistent and structured training [30]. Therefore, we consider it unlikely that the moderate physical activity routines during this early adjustment period had a substantial influence on the outcomes. Future research should aim to include a more diverse population of individuals with developmental disorders and adopt longitudinal or interventional designs to examine the effectiveness of exercise support programs and to develop practical strategies for implementation.

## Conclusions

This study evaluated cardiorespiratory endurance in adolescent and young adult males aged 15 to 20 years with developmental disorders by conducting maximal exercise tests to measure VO_2_peak. The findings revealed that both absolute and relative VO_2_peak values were markedly lower than the normative values previously reported for typically developing males of the same age group. To our knowledge, this is the first study to report directly measured VO_2_peak using maximal exercise testing in this population. These results may serve as a reference for future research in the field.
